# Use of Gamified Digital Tools in Daily Tasks of Health Care Workers: Scoping Review

**DOI:** 10.2196/70480

**Published:** 2025-10-20

**Authors:** Binita Paudel, Hussein Al-Shehabi, Rita Dörner, Charbel El Bcheraoui, Tessa Lennemann, Andrea Bernasconi

**Affiliations:** 1 Evidence-Based Public Health Unit, Centre for International Health Protection, Robert Koch Institute, Gricht Strasse 27, Berlin, 13347, Germany, +4930187545067; 2 Institute of International Health, Charité – Universitätsmedizin Berlin, Berlin, Germany; 3 Deutsche Gesellschaft für Internationale Zusammenarbeit (GIZ), Bonn, Germany; 4 Unité Epidémiologie et Recherche Clinique, Réseau de l’Arc, Saint-Imier, Switzerland

**Keywords:** game elements, gamification, gamified digital tools, health care workers

## Abstract

**Background:**

The quality and effectiveness of health care service delivery are significantly influenced by the engagement and motivation of health care workers. Integrating gamified digital tools (GDTs) into health care workers’ workflows presents a promising approach to enhancing them. However, there is currently a lack of evidence supporting the implementation of such interventions.

**Objective:**

This scoping review aims to summarize existing evidence on the influence of GDTs on the daily tasks of health care workers.

**Methods:**

A scoping review was conducted following the PRISMA-ScR (Preferred Reporting Items for Systematic Reviews and Meta-Analyses extension for Scoping Reviews) guidelines for scoping reviews. We conducted a comprehensive search across different databases (PubMed, EMBASE, *International Journal of Serious Games*, Cochrane Library, and Google Scholar) for peer-reviewed studies and (OpenAlex, GreyNet, and IEEE Xplore) for gray literature published between January 2010 and January 2024. Eligibility criteria, developed using the SPIDER (Sample, Phenomenon of Interest, Design, Evaluation, and Research) framework, included qualitative, quantitative, and mixed methods studies involving health care workers using GDTs for daily tasks. Studies in English, French, Spanish, or Italian were eligible. Keywords and medical subject headings related to gamification and health care workers were used. The studies were screened, eligibility was assessed, and data were extracted. A narrative synthesis was used to summarize and interpret the findings.

**Results:**

Of 5844 studies, 12 met the inclusion criteria and were included in the analysis. These studies exhibited considerable heterogeneity in the application of gamification. Feedback, competition, and dashboard features were the most common gamification elements identified. The implementation of these elements led to enhanced engagement, increased motivation, improved task completion, and promoted healthy competition among staff across various health care settings.

**Conclusions:**

Integrating GDTs into health care workers’ tasks holds significant potential to enhance engagement and motivation. However, empirical evidence is still lacking. Comparative studies are needed to gain comprehensive insights into the benefits and limitations of gamification in health care.

## Introduction

The quality and effectiveness of delivering health care services greatly depend on the engagement and motivation of health care workers (HCWs), as their willingness to perform tasks in the workplace directly influences the quality of care offered [1,2]. Surveys conducted in several countries indicated a lack of motivation as the second most critical issue within the health care workforce, preceded only by the problem of staff shortage [2]. Many daily tasks performed by HCWs, such as medical data collection or routine physical examinations, are particularly prone to motivation lapses. Yet, these tasks are essential for generating reliable data that underpin public health decisions, improving evidence-based patient management, and, consequently, delivering effective and high-quality health care [3,4].

The growing integration of information and communication technologies (ICTs) into the health care sector has increasingly supported HCWs in clinical practice. According to the World Health Organization, by 2021, 80% (n=120) of member states had developed national digital health strategies [5]. Furthermore, global spending on digital health was estimated at approximately USD 288 billion in 2024, largely driven by investments in telemedicine, artificial intelligence, and mobile health (mHealth) solutions [6].

The advent of ICT presents an opportunity to address staff motivation through gamified digital tools (GDTs). GDTs are software applications or platforms that integrate game mechanics, such as points, badges, levels, leaderboards, or challenges, into nongame contexts to enhance user motivation, engagement, and learning outcomes [7].

The term “gamification” was first introduced in 2002 to enhance the learning process in the consumer goods industry by incorporating playful elements into software [8-10]. The concept of gamification has gained popularity in recent years [11]. Defined by Deterding et al [12] as “the incorporation of game design elements into nongame contexts,” gamification entails integrating game-like elements, such as points and leaderboards, into environments that traditionally do not fit within the conventional scope of gaming [13]. For Zichermann and Cunningham [14], gamification is “the process of game-thinking and game mechanics to engage users and solve problems*,*” while Bunchball [15] describes it as applying game elements to nongame activities, aiming to alter people’s behaviors. Bunchball also recognizes gamification as a powerful strategy for influencing and motivating various groups of people, such as children, adults, or workers [15]. In fact, the primary purpose of gamification is to encourage positive behavior in individuals while also enhancing their motivation and participation in specific activities [16]. Thus, gamification has the potential to motivate employees while performing monotonous and repetitive tasks, boost their involvement, foster a positive approach to their jobs, and, as a result, elevate their overall productivity [17].

The application of gamification is today widespread across various sectors, including education, business, marketing, and health care. In education, gamification can make learning activities more engaging and motivate students to achieve their goals [18,19]. For instance, Duolingo, one of the most common mobile-assisted language learning platforms, incorporates numerous game-like elements to enhance students’ engagement and motivation to learn new languages [20], and Kim et al [21] reported that college students in a gamified class outperformed their counterparts in traditional lecture-based classes by 40%. In business and marketing, gamification encourages employees to complete tasks or increase customer engagement with a product or service [22]. In health, gamification strategies are designed to motivate patients to follow treatment plans to maintain healthy lifestyles [23]. “Heart Game” [24] and “Heart Health” [25], which are gamified disease self-management apps for cardiovascular health, are examples of these health behaviors change support systems [11]. Along with the high penetration of smartphones and mobile devices into society [26], gamification has the potential for substantial growth. Its market, estimated at USD 15.4 billion for 2024, is expected to reach USD 48.7 billion by 2029 [27].

The basic structure of any gamification approach is the “gamification element*.*” A gamification element refers to a specific feature or component derived from games that are used to enhance engagement, motivation, and participation in nongame contexts. These elements are incorporated into various activities or systems to make them more appealing and enjoyable. Examples of game elements are points, badges, leaderboards, and rewards [28]. Game elements like badges, feedback, and achievements foster a sense of competence, while the inclusion of teams and social networks enhances feelings of connectedness. Tailored features, such as avatars, empower individuals by providing control and choice over their actions, thereby reinforcing their sense of autonomy [29,30]. A comprehensive categorization of gamification elements was compiled by reviewing several authors’ works [16,28,31-40], and it is provided in Table 1.

**Table 1.** A comprehensive list of gamification elements.

Elements of gamification	Description
Badges/trophies	A visual symbol representing a predetermined accomplishment and acknowledgement
Feedback	Immediate feedback on users’ performance and achievements which induces users’ sense of progress and achievement during tasks, increasing awareness of their own progress and enhancing their experiences
Competition	Team-based competition encourages users to develop the vested interest in the task, thereby sustaining their engagement and involvement throughout the activity. A set of tasks that incentivize who finishes early and quickly
Time pressure/countdown timer/ clock/deadlines	Time itself used to pressurize the users to finish a task, thereby enhancing engagement and challenging users within a defined timeframe
Leaderboards/scoreboard/ dashboard/rank	A leaderboard in gamification motivates competition and fosters engagement by showcasing and ranking participants based on their performance
Rewards	Serve as incentives, driving user motivation and fostering continued engagement by acknowledging and reinforcing positive actions
Avatar	Avatars in gamification allow individuals to represent themselves. Digital self-representation of users
Progress	Progress in gamification helps to track the progress made thus far, assess achievements and select desired badges or trophies, providing a clear overview of accomplished milestones and guiding future goals
Level	Levels represent the position of users within tasks, with each completed task potentially advancing them to the next level, introducing new challenges and difficulties
Points/scores	A quantification of achievements following the successful completion of tasks and responsibilities
Quest	A quest in gamification refers to the task or challenge that motivates users to engage with the application or system
Notification	Notifications prompt users to perform desired activities
Narrative story	A narrative story creates a scenario or theme inspired by a gamified system

Recent studies have increasingly employed structured taxonomies to categorize gamification mechanics in different domains, emphasizing the importance of aligning specific game elements with contextual objectives. For instance, Hervás et al [41] proposed a taxonomy comprising 6 fundamental categories tailored to the health sector, particularly aimed at fostering behavioral change. These categories are goals (direction and purpose), status (recognition via points or badges), randomness (surprise through chance), appointment (timed engagement), progression (tracking advancement), and social (interaction and competition). Schmidt-Kraepelin et al [42] also developed a taxonomy of gamification concepts for health apps. This taxonomy comprises 12 dimensions, each characterized by 2-3 mutually exclusive attributes. Toda et al [43] later presented a gamification taxonomy in an educational environment and identified 5 different categories: performance (acknowledgment, level, points, progression, and stats), ecological (chance, choice, rarity, and time pressure), social (competition, cooperation, reputation, and social pressure), personal (novelty, purpose, challenges, renovation, and sensation), and fictional (narrative and storytelling).

The mechanism behind gamification could be simplified into extrinsic and intrinsic motivations. Extrinsic motivation refers to the use of external rewards or incentives to encourage people to engage in certain behaviors or actions. In contrast, intrinsic motivation is driven by internal rewards such as personal satisfaction or enjoyment [28].

Despite the widespread success of gamification in promoting learning behaviors, advancing health education [44], and promoting healthy habits [45], the extent of its effectiveness in enhancing the activities of HCWs is still underexplored. This gap persists even amid the rapid and ongoing digital transformation of health systems worldwide. While ICT enhances efficiency by reducing administrative burden and improving clinical decision-making, its adoption could present challenges as frontline HCWs must navigate complex digital interfaces, manage large volumes of patient data, and adapt to evolving systems. Poorly designed tools can lead to cognitive overload, workflow disruptions, and increased stress, impacting job satisfaction, burnout rates, and, finally, patient care quality [46-48].

This study aims to bridge this knowledge gap by examining how GDTs could shape the daily work experience of HCWs, ensuring that technological advancements enhance rather than hinder their ability to provide high-quality care.

Through this unique focus, this study can provide evidence of gamification in HCW’s environment and practical insights for policymakers, health care institutions, and digital tool developers.

This review is the first to explore the integration of GDTs into the daily workflows of HCWs and to present evidence of their influence on their daily tasks.

## Methods

For this review, we adopted the PRISMA ScR (Preferred Reporting Items for Systematic Reviews and Meta-Analyses extension for Scoping Reviews) [49] (Multimedia Appendix 1).

### Search Strategy and Selection Criteria

We conducted a comprehensive literature search, utilizing various databases and search terms. PubMed, EMBASE, *International Journal of Serious Games*, Cochrane Library, and Google Scholar were searched for peer-reviewed research, while OpenAlex, GreyNet, and IEEE Xplore were searched for gray literature known to index technical reports, conference proceedings, and non-peer-reviewed research relevant to gamification. Our search strategy included Medical Subject Headings and keywords such as “gamification,” “videogame,” “healthcare worker,” and “healthcare professional” to ensure the inclusion of all relevant studies from both peer-reviewed and gray literature. We employed Boolean operators like “OR” and “AND,” along with truncation (*), to account for variation in word endings, such as gamif* for gamification or gamified. An expert librarian supported this rigorous search to ensure the inclusion of all relevant studies. The detailed search strategy is available in Multimedia Appendix 2. After an initial screening based on titles and abstracts, we conducted a second screening on the full text retrieved by 2 authors (BP and HAS) through Rayyan [50], with a third author (AB) consulted in case of disagreement. The weighted kappa coefficient of Cohen was calculated to assess the degree of agreement between the 2 reviewers, and it was applied as suggested by Landis and Koch [51]. Additionally, we conducted a supplementary manual search through the reference lists of the included studies.

### Eligibility Criteria

The SPIDER (Sample, Phenomenon of Interest, Design, Evaluation, and Research) framework [52] determined the inclusion and exclusion criteria as outlined in Table 2.

**Table 2.** Eligibility criteria according to the SPIDER (Sample, Phenomenon of Interest, Design, Evaluation, and Research) framework.

Criterion	Inclusion	Exclusion
Sample (S)	Health care workers including community health workers, community health volunteers, and data clerks	Patients, students, non-health care workers
Phenomenon of interest (PI)	Gamified digital tools considered on the basis of the elements of gamification embedded (points, badge, feedback, etc) used in the daily activities	Gamified tools not involving health care delivery or lacking gamification features
Design (D)	Peer-reviewed sources and gray literature	Informal sources without clear institutional affiliation or academic relevance
Evaluation (E)	Studies reporting on tool features, usability, engagement, health care worker experience, or outcomes related to work performance	
Research type (R)	Qualitative, quantitative, mixed methodology	Editorial, commentaries, editorials, opinion pieces, or protocols
Others		
Publication year	Published between January 2010 and January 2024	
Language	English, French, Italian, Spanish	Publications in other languages
Technology format	Tools applying gamification through ICT^a^ (eg, mobile apps, electronic health records, telemedicine platforms)	Tools not using ICT (eg, physical board games)
Publication type	Peer-reviewed journal articles, conference papers, and abstracts	Unpublished material, blogs
Subject focus	Gamified digital tools used for health care service delivery or operational/clinical support tasks	Tools used solely for training, disease self-management, or in non-health care contexts
Gamified tool user group	Health care workers as direct users of the gamified digital tool	Tools used exclusively by patients, students, or non-health care professionals

a ICT: information and communication technology.

Only interventions involving GDTs for daily health care tasks were selected for inclusion, provided they featured at least 1 gamification element. For this review, HCWs were defined broadly to include not only clinical staff but also volunteers, data clerks, and health administrators who used GDTs in their daily tasks, for example, by entering clinical data into databases within a health care setting. Studies in which gamification elements were not implemented within ICT (eg, board games) were omitted, as well as studies in which GDTs were primarily aimed at training, education, and self-care disease management, or not related to health care.

In our study, we considered studies published between January 2010 and January 2024, as the term gamification gained widespread recognition after 2010 [10-12], and to capture the progression of digital health since the release of the “Principles for Digital Development“ [53]. Additionally, studies of all designs (eg, qualitative, quantitative, and mixed methods studies) published in English, French, Spanish, or Italian were included.

### Data Extraction and Quality Appraisal

One author (BP) conducted independent data extraction using a spreadsheet prepared by consensus by the reviewers using Microsoft Excel [54], subsequently revised by 2 authors (AB and HAS). For each included study, we extracted information on the year of publication, country of implementation, study objective, design, data collection method, target population, sample size, type of digital intervention, application context, gamification elements used, and main findings.

Two authors (BP and AB) conducted the quality assessment. A third author (HAS) was consulted in case of disagreement. The quality assessment was based on the method outlined by Sardi et al [11], which involved utilizing a set of five closed-ended questions to assess the relevance and quality of the publication: (1) whether the paper provides a detailed description of the game elements employed, (2) if the study presented empirical results, (3) if the benefits and (4) limitations of gamification were explicitly addressed, and (5) the relevance of the source where the study was published investigated through Scimago Journal & Country Rank [55]. The score assigned to each study could range from 0 (lowest) to 6 (highest). Based on the average score, the studies were classified into 3 categories: high (5 or above), average (below 5 and above or equal to 3), and poor (below 3).

The quality assessment checklist and results are given in Multimedia Appendix 3. To assess the degree of agreement between the raters, a weighted kappa coefficient of Cohen was calculated.

### Data Synthesis

Given the heterogeneity of the studies, we employed a narrative approach to summarize the findings and address our research question. As GDTs have been integrated into various digital health interventions, a classification based on the different working contexts and the professional background of health staff was deemed most suitable for conceptualizing and describing their application. This format also aligns with the notion that digital tools should be designed according to the profiles of their users, as stated by the “Principles for Digital Development” [53]. We, therefore, classified users into three main categories: (1) community health care workers (CHWs), frontline workers engaging with communities outside of health facilities; (2) HCWs working in primary health care (PHC); and (3) HCWs working in secondary health facilities, such as hospitals. Finally, as suggested by several authors [11,16,40,56-59], we summarized the benefits of the GDTs on the HCWs as follows: increased engagement, enhanced motivation, task completion and behavior change, and heightened healthy competition.

Increased engagement refers to HCWs’ higher involvement and commitment to their tasks, leading to improved performance and better patient care [11,56-58]. Enhanced motivation means a stronger drive to perform duties effectively, resulting in higher job satisfaction and productivity [11,16,56-58]. Task completion involves the consistent and timely fulfillment of assigned duties based on a behavior change [56,59]. Healthy competition is a positive rivalry that encourages staff to excel, share best practices, and support each other’s growth, enhancing overall care quality [40].

## Results

### Overview

A total of 5844 records were identified through a comprehensive search of multiple databases. After removing 554 duplicates, 5290 studies were screened by title and abstract. Of these, 4784 were excluded for not meeting the study objectives, primarily because they did not address gamification. An additional 195 studies were excluded for not focusing on health care, and 29 were removed based on publication type (eg, editorial or commentary). Subsequently, 506 studies were sought for retrieval. Of these, 6 could not be accessed as full text and were therefore excluded. The final 500 studies underwent a second screening. At this stage, 488 were excluded: 195 focused on patient-centered intervention rather than HCWs, 158 did not incorporate gamified components, 89 described gamified tools solely for training and educational purposes, 29 included gamification elements not integrated into a digital platform, and 17 involved GDTs not intended for use by HCWs. Finally, based on the eligibility criteria, 12 studies were selected for the final review, comprising 9 studies that had been peer-reviewed and 3 Congress proceedings.

**Figure 1.**
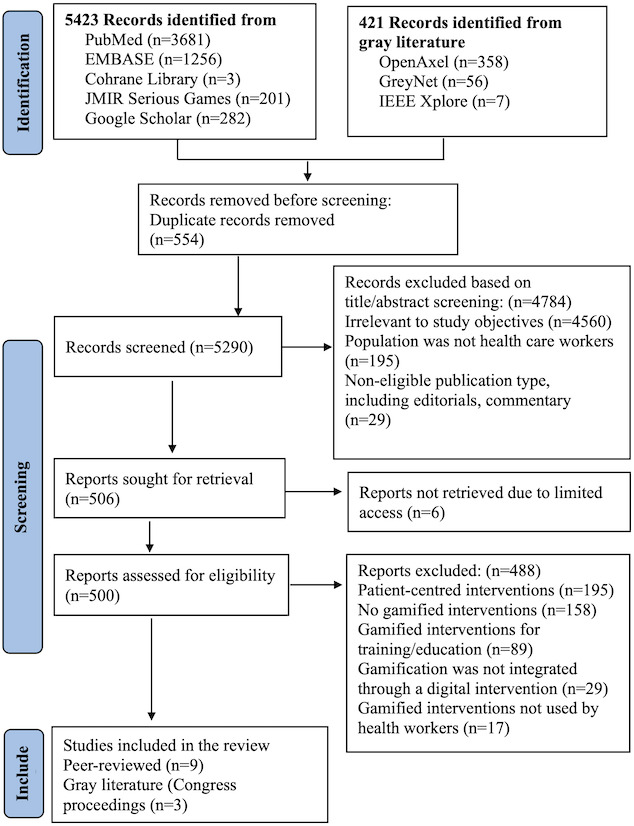
PRISMA (Preferred Reporting Items for Systematic Reviews and Meta-Analyses) flow diagram illustrating the study selection process.

The interrater reliability for selecting the included studies, measured using Cohen’s weighted kappa coefficient [51], was 0.72, suggesting a substantial agreement between raters. The PRISMA flow diagram of the study selection process is presented in Figure 1.

### Quality of the Included Studies

The average quality score of the selected studies, based on the assessment method proposed by Sardi et al [11], was 3.4, with scores ranging from 1 (minimum) to 5 (maximum). A total of 5 studies were classified as high quality, 4 as average quality, and 3 as poor quality. Most of the studies (n=10, 83.3%) lacked a discussion concerning the limitations of the GDTs, and many (n=5, 41.7%) did not discuss any benefit. A total of 8 studies (66.7%) were published or presented in recognized journals or Congresses. The Cohen weighted kappa coefficient among the raters was very good: 0.83 [51].

### Basic Information of Included Studies

Of the 12 included studies, 9 (75%) were peer-reviewed in scientific journals, while the remaining 3 (25%) were sourced from gray literature (Congress proceedings). A total of 6 (50%) studies originated from high-income countries (HICs): Canada [60], Australia [61], the United States [62-64], and Portugal [65]; 6 (50%) from low- and middle-income countries (LMICs): Nigeria [66], India [67], Pakistan [68], Kenya [69], and Ethiopia [70]. One study was a multi-country intervention carried out in Bangladesh, Burkina Faso, and Ecuador. A total of 3 interventions focus on CHWs’ use of GDTs, 3 are implemented in PHC settings, and 6 in hospitals. The majority of the studies was published before 2020 (n=10, 83.3%). The most common study design was quasi-experimental (n=5, 41.7%), followed by qualitative studies (n=2, 16.7%). Of the remaining 5 studies, 1 was a cross-sectional study, 1 a case study, 1 an interventional pilot study, 1 a diagnostic accuracy study, and 1 an observational study. The characteristics of each study are presented in Table 3.

**Table 3.** Overview of the characteristics of the included studies.

Author Year Country/s	Objectives	Study type Sample size	Digital intervention	Health domain or application context	Results	Context
Luedtke et al [66] 2023 Nigeria	to improve SAP^a^ compliance to guidelines	Interventional Surgeons (n=20)	Surgeons received real-time feedback from a virtual mentor concerning the most appropriate SAP	SAP	321 SAP prescriptions recorded. 12% adjusted to guidelines after app feedback (*P*<.01). 10% changed decision on SAP necessity (*P*<.01)	Hospital
Finette et al [71] 2019 Bangladesh Burkina Faso, Ecuador	To improve care quality by enhancing iCCM^b^ protocol compliance	Validation study FLWs^c^ (n=49), LHPs^d^ (n=22), Children aged 2-60 mo (n=861)	The platform guides FLWs through clinical assessments with embedded demos and animated GIFs^e^ to improve data quality	iCCM	FLW assessments with the tool showed 84%‐99% specificity correlation with experts; triage recommendations also aligned. The tool was appreciated for usability, learning, and support	FLWs
Shah et al [67] 2019 India	To assess the uptake, feasibility, effectiveness, and perceptions of a mobile phone app. To increase the coverage of maternal, newborn, and child health services	Mixed methods study Mothers of 1‐ to 4-month-old infants (n=99), Mothers with 6- to 9-month-old infants (n=187), FLWs (n=15)	FLWs (ASHAs^f^) register pregnant women and children via the app, which schedules home visits with reminders and urgency indicators. Checklists and algorithms support diagnosis and treatment. A web interface enables real-time supervision and tracks high-risk cases, inventory, and incentives	Mother and child health care	Coverage improved in the intervention area versus control for newborn care (56% vs 10%), exclusive breastfeeding (44% vs 23%), and care-seeking for maternal (77% vs 57%), neonatal (78% vs 27%), and pneumonia screening (41% vs 24%). FLWs showed sufficient app competency with an 88% login rate during the study	FLWs
Zaidi et al [68] 2020 Pakistan	To improve data quality in EPI^g^	Qualitative study Vaccinators (n=26), government district managers (n=7)	Vaccinators use an app to record data, track stocks, and send schedule reminders. Real-time data informs monthly meetings, supports performance recognition, and promotes best practices	EPI	Digital immunization tracking was well-received; data were more valid than manual entry. Key motivators: ease of use, data quality, recognition, field support, and empowerment	PHC^h^
Mckeown et al [60] 2016 Canada	To improve sepsis identification and management by increasing guidelines compliance	Quasi-experimental study Clinicians in emergency departments (n=not stated)	The “150 Lives in 150 Days” campaign engaged clinicians in adopting sepsis protocol. Patient data were collected via apps, websites, or papers. Game-like elements (countdown, lives saved) created urgency, while feedback and learning modules boosted engagement	Sepsis identification and management	Severe sepsis mortality dropped to 6.4% (Oct 2013-Mar 2014) from 21.1% in earlier periods	Hospital
Orchard et al [61] 2019 Australia	AF^i^ screening in general practice	Qualitative study GPs^j^ (n=21), nurses (n=13), practice managers (n=11)	The app used EMRs^k^ to guide evidence-based AF treatment per guidelines. Feedback to practices covered screening totals, results, AF diagnoses, and treatment compliance	AF screening	Users appreciated the tools, though technical issues occasionally disrupted screening. Time constraints were the main barrier	PHC
Orwoll et al [62] 2018 USA	To prevent CLABSI^l^	Quasi-experimental Nurses (n=105)	During shifts, nurses use the app for line care and to track protocol adherence and performance. A gamification engine enables team competitions	CLABSI prevention	Compared to the previous year, CLABSI rates in the intervention group (9886 line-days) dropped by 48% (*P*=.03), with no significant change in controls (7879 line-days)	Hospital
Wenner et al [69] 2014 Kenya	To design an intuitive, fun, and efficient interactive tool for data collection	Case study FLWs (n=not stated)	Intuitive inputs (sliders, dials, buttons) support valid data entry, while visual cues (eg, height slider with tall/short images) enhance clarity. Sensory feedback (lights, sounds) creates an engaging, accessible user experience	Data collection	Leveraging tangible computing reduces the technical literacy required to use a device, crucial in low-resource settings	FLWs
Little et al [70] 2013 Ethiopia	To support HCWs^m^ and midwives in mother and child health care service	Mixed method: HCWs (n=20), midwives (n=12)	An analytics dashboard and mobile scorecard enable health workers and supervisors to monitor their progress and key performance indicators	Mother and child health care	HCWs quickly adapted to touchscreens with minimal support. Gamification fostered ownership and empowerment	PHC
Owens et al [64] 2018 USA	To reduce delay in data collection	Quasi-experimental study HCWs (n=not stated)	A computer app calculates delays from daily EMR extracts, displaying a real-time color-coded dashboard to alert staff about patient delays	Data collection	Patient delays were reduced by 27% in the >60-min category	Hospital
Collins et al [63] 2013 USA	To measure the impact of an automated hand hygiene monitoring system	Quasi-experimental study HCWs (n=60)	Hand hygiene programs focusing on solution dispensing, compliance, and infection rates, featuring team competition with weekly compliance graphs by unit, and individual feedback through point-of-care units and posted compliance reports	Hand hygiene compliance	75.4% increase in total hand hygiene solution dispensing, 96.4% improvement in monthly hand hygiene compliance, and health care–associated infections reduction by 11.1%	Hospital
Marques et al [65] 2017 Portugal	To improve hand hygiene compliance	Cross-sectional study Nurses (n=6)	Real-time data collected and feedback from an indoor location system regarding each nurses’ hand hygiene compliance	Hand hygiene compliance	The impact of this intervention is still under evaluation	Hospital

a SAP: surgical antibiotic prophylaxis.

b iCCM: Integrated Community Case Management.

c FLW: front line worker.

d LHP: local health care professional.

e GIF: graphics interchange format.

f ASHA: Accredited Social Health Activist.

g EPI: Expanded Programme on Immunization.

h PHC: primary health care.

i AF: atrial fibrillation.

j GP: general practitioner.

k EMR: electronic medical record.

l CLABSI: central line-associated bloodstream infections.

These 12 studies address a variety of health topics, including compliance with hand hygiene [63,65], mother and childcare [67,70], health-related data collection [64,69], surgical antibiotic prophylaxis (SAP) [66], Integrated Community Case Management (iCCM) [71], and Expanded Programme on Immunization (EPI) implementation [68], sepsis diagnosis and therapy [60], atrial fibrillation (AF) screening [61], and central line-associated bloodstream infections prevention [62]. Table 3 presents a comprehensive overview of the included studies.

The most common gamification elements integrated into the GDTs were the use of feedback (n=9, 75%), use of competition (n=6, 50%), leaderboards/dashboards/scoreboards (n=6, 50%), badges (n=3, 25%), and time pressure elements (n=3, 25%). A total of 3 (25%) digital interventions [67,69,71] incorporated a single gamification element, whereas most (n=9, 75%) integrated at least 2. Table 4 presents a summary of the elements of gamification adopted in these studies.

**Table 4.** Elements of gamification presented in the reviewed studies.

Elements of gamification/studies	Luedtke et al [66]	Finette et al [71]	Shah et al [67]	Zaidi et al [68]	Mckeown et al [60]	Orchard et al [61]	Orwoll et al [62]	Wenner et al [69]	Little et al [70]	Owens et al [64]	Collins et al [63]	Marques et al [65]
Feedback	✓^a^	✓		✓	✓	✓		✓	✓		✓	✓
Badges	✓				✓							✓
Time pressure/countdown clock			✓		✓					✓		
Competition				✓	✓	✓	✓				✓	✓
Narrative or story					✓							
Challenges	✓				✓							
Progress					✓							
Leaderboard/scoreboard/dashboard					✓		✓		✓	✓	✓	✓
Award					✓		✓					
Social networking					✓		✓					
Points							✓					✓
Level												✓
Virtual goods/rewards/ prize					✓							✓
Cooperation												✓
Emotional relationship												✓

a ✓: elements of gamification presented.

### Use of Gamified Digital Tools by Community Health Workers

We identified 3 studies [67,69,71] that involved CHWs using GDTs, all conducted in LMICs.

One intervention in Kenya focuses on improving data collection by introducing a custom-made GDT device with intuitive input mechanisms like sliders and dials, complemented by visual aids and sensory feedback (eg, when collecting height data, the device featured images of a tall individual at the top and a shorter individual at the bottom of a height slider). This approach was reported to enhance CHWs’ engagement, but it does not present any evidence of increased effectiveness in data collection [69].

In India, a quasi-experimental study evaluated an intervention with a smartphone app for health activists and a web interface for PHC staff to improve maternal and childcare. The app used time pressure as a gamification element, turning text red for overdue tasks to prompt faster completion. The intervention increased home-based newborn care from 10% to 56%, exclusive breastfeeding from 23% to 44%, care-seeking for maternal and neonatal complications from 57% to 77% and 27% to 78%, respectively, and pneumonia care from 24% to 41%. The time pressure likely improved task completion, though the study did not quantify its specific impact [67].

MEDSINC, a mobile health platform, integrates Integrated Community Case Management guidelines to support CHWs in the clinical assessment, triage, and treatment of children aged between 2 months and 6 years. This tool includes demonstrations and animated graphics interchange formats to enhance data collection quality and accuracy. Children’s clinical assessments by CHWs using MEDSINC showed a specificity correlation of 84%-99% with local health care experts, and their triage recommendations were highly correlated [71]. The tool received positive feedback for its user-friendliness, educational value, and enhancement of job performance, showing that gamified data visualization can motivate data collection.

### Use of Gamified Digital Tools in Primary Health Care

A total of 3 interventions evaluated GDTs in PHC: two in LMICs and one in an HIC [61,68,70].

An observational study in Ethiopia [70] evaluated an Open Data Kit platform for maternal and childcare case management. The tool included an analytic dashboard and a scorecard to monitor key performance indicators. The intervention was well-received by HCWs, who quickly adapted to smartphones and required minimal technical support. However, while visualizing progress appeared to motivate HCWs’ performance, the study did not establish a causal link between the heightened motivation and the overall impact of the intervention.

In Pakistan, an app was developed to monitor routine immunization by recording and uploading data, synchronizing profiles with central databases, and managing vaccine inventories [68]. The tool used real-time data to monitor vaccinations, track incomplete cases, plan outreach, adjust micro plans, and allocate fuel allowances. It demonstrated greater accuracy than manual data entry and used gamification to highlight top performers (leaderboard), reward high achievers, and foster healthy competition. Also, individual vaccine performance feedback increased vaccinators’ engagement.

A qualitative study investigated the use of an eHealth tool within an atrial fibrillation screening program in PHC in Australia [61]. The app extracted EMR data to provide real-time screening prompts and treatment recommendations. Additionally, a smartphone electrocardiograph tool was used for diagnosis. The app generated stroke risk scores and treatment advice, with monthly individual de-identified data reports for quality improvement. These reports included screening rates, new diagnoses, and treatment adherence. HCWs appreciated the eHealth tools, and regular feedback was beneficial for quality improvement, increased the proportion of eligible people to be screened for atrial fibrillation, and maintained motivation. Moreover, the feedback encouraged app usage by fostering healthy competition.

### Use of Gamified Digital Tools in Hospital Settings

A total of 6 interventions were carried out in hospital care, 5 [60,62,63,65,66] focusing on reducing health care–associated infections by increasing adherence to guidelines and procedures, and 1 study [64] was carried out on patient flow management.

Two interventions [63,65] incorporated gamification elements to improve hand hygiene in intensive care units. In the United States, a multifaceted strategy included hand washing automated monitoring machines, communication, and leadership initiatives [63]. Key features included hospital-wide competitions, public compliance postings, and electronic point-of-care performance reporting. This approach led to a 75% increase in hand hygiene solution dispensing, a 96% improvement in hand hygiene compliance, and an 11% reduction in health care–associated infections, demonstrating that individual feedback and team competition can significantly impact health outcomes.

A similar intervention in Portugal used an automated monitoring system based on indoor location detection to assess nurses’ proximity to patients and handwashing habits. Initial trials faced accuracy issues, preventing a full evaluation of the gamification elements. However, the intervention aimed to provide real-time feedback through dashboards, email notifications, and competitions with avatars and leaderboards, with its impact on hand hygiene compliance still under evaluation [65].

To prevent nosocomial infections, another study evaluated the use of the CLABSI (Central Line-Associated Bloodstream Infections) app to engage nurses in preventing infections caused by central catheters in intensive care units through a quasi-experimental design [62]. This app allowed nurses to monitor protocol compliance and compare their performance against benchmarks and their units. Over 12 months, the intervention group showed a 48% decline in CLABSI rates compared to the previous year, while the control group showed no significant change. The app included gamification elements such as leaderboards, social networking, points, team competitions, awards, and prizes. A total of 3 competitions were held during the study period. Self-assessment completions were significantly higher, with a median of 43 self-assessments per unit per month versus two in non-contest months. Nurse engagement with the app was also 10 times higher during contest months.

Prevention was at the core of another study conducted in Nigeria, aiming to change surgeons’ behavior regarding SAP [66]. A virtual mentor provided real-time feedback on surgical risk, antibiotic choice, and therapy duration. A WhatsApp group supported app usage, and 321 surgeries were recorded. The study found that 12% (n=37) of surgeons adjusted their decisions to align with guidelines, and 10% (n=33) changed their antibiotic prophylaxis decisions. Gamification elements such as challenges and digital badges increased engagement, though the app’s lack of EMR integration limited its effectiveness.

In 2015, Canada launched a 150-day campaign to enhance sepsis treatment in emergency departments using a gamification approach to increase the adoption of sepsis protocol [60]. Clinicians organized themselves as teams, and clinical data from sepsis patients were collected online, via the app, or on paper and reviewed for protocol adherence. The campaign featured a countdown clock, a daily tally of saved lives, awards, and a feedback module. Positive extrinsic motivators, such as feedback and rewards, along with intrinsic motivators, including mastery, autonomy, relatedness, and purpose, were incorporated into this life-saving campaign, which showed a reduction in severe sepsis mortality from 21% to 6%. The healthy competition among clinicians was crucial to the campaign’s success.

Lastly, 1 US-based intervention was not strictly connected to clinical care but focused on patient flow management. A radiology department implemented a tailored dashboard to address patient data collection delays. This application calculated delays using daily EMR data and displayed real-time, color-coded dashboards to alert staff. A quasi-experimental study showed that the intervention reduced patient delays over 60 minutes by 27%, with time pressure as the gamification element [64].

## Discussion

### Principal Findings

The primary purpose of this review was to collate existing evidence on how incorporating GDTs into the daily workflow of HCWs affects their behavior and performance in health-related tasks. Of the 12 reviewed studies, 5 [60,62,66,68,69] reported that integrating different game elements such as challenges, prizes, badges, feedback, leaderboards, social networks, competition, and time pressure helps to enhance HCWs’ engagement; 3 [60,61,71] emphasized the role of gamification in increasing motivation; 3 [62,66,67] promoted task completion and behavior change; and 4 [60,61,63,68] boosted healthy competition among users.

The reviewed studies suggested that GDTs can improve hand hygiene compliance, sick child assessment, maternal and child health care, sepsis management and control, and CLABSI rates. However, we observed significant heterogeneity across the interventions, due to differences in health care settings, the purposes of the digital tools, and user characteristics. These differences make it challenging to identify a consistent impact of GDTs. Furthermore, gamification was rarely the primary focus of the research, as most studies prioritized a comprehensive evaluation of the broader ICT strategy. As a result, isolating the specific effects of gamification from the overall digital intervention remains difficult, limiting the ability to establish clear causal links.

In other contexts, literature is rich with contributions from authors who highlighted the positive effect of gamification on education and learning [44,72,73], as well as health and wellness [45]. However, to the best of our knowledge, this review is the first to explore the integration of GDTs into the daily workflows of HCWs.

The reviewed studies offer several important insights. Evidence from HICs suggests that GDTs are most effective when integrated into a broader organizational framework that includes workflow reorganization and improved intra-team communication. Additionally, the integration of EMRs plays a key role by enabling real-time access to patient data, thereby facilitating more effective performance feedback and the delivery of timely clinical prompts. However, these structural elements require continuous attention to ensure seamless integration and to maximize the impact of GDTs, particularly when they are strategically incorporated into comprehensive public health initiatives.

On the other hand, for HCWs with limited digital literacy, particularly in LMICs, the reviewed studies suggest that a simple and engaging user interface plays a particularly important role in facilitating adoption and sustained use. Many CHWs may face challenges navigating complex systems, making it essential for the interface to be intuitive, visually appealing, and engaging. Poorly designed or overly complicated interfaces can act as significant barriers to adoption and may ultimately undermine the effectiveness of the intervention.

Among the 12 studies reviewed, only 2 [65,69] explicitly addressed the challenges associated with integrating gamification elements into broader ICT interventions. One study noted that incorporating gamification elements, which are visual and acoustic, into a digital data collection tool can drain the device’s power rapidly, potentially reducing its appeal [69]. Another study in Portugal found that requiring HCWs to engage with gamification elements like badges, virtual goods, and content unlocking outside working hours decreased their enthusiasm [65]. The same study suffered significant setbacks due to technological issues, which hampered an evaluation of the benefits of gamification.

The difficulties encountered in these studies exemplify the importance of careful design in GDTs: a technologically flawed product can compromise all the benefits of gamification, and a solution not designed with users in mind can lead to resistance from them. Researchers and developers must ensure that the gamification elements are relevant and engaging for diverse user groups, as many factors can influence attitudes toward GDTs, including age, education level, work experience, culture, and computer skills [74]. Although gamification design is fundamentally user-centered [33], none of the interventions reviewed mentioned involving users in the design process.

### Comparison to Prior Work

In this review, among the various gamification elements utilized and embedded in digital health interventions, feedback emerged as the most commonly used component. This is consistent with previous reviews on the use of gamification in other domains [11,75]. Sardi et al [11] and Edwards et al [75] reported that feedback was used in over 90% (n=60) of the studies they reviewed on eHealth and health promotion, respectively. The present review also highlights that performance feedback enhances HCWs’ experience and engagement in their tasks, leading to behavioral change [40,75].

Competition emerged as the second most utilized element of gamification in 50% (n=6) of the included interventions, corroborating findings from a previous systematic review on gamification for health profession education [18]. Dashboards, leaderboards, and scoreboards were the most commonly used elements to convey feedback and foster competition. However, the use of external rewards, such as feedback, competitions, and leaderboards, to motivate HCWs can present several important limitations, as suggested by several studies. Although such incentives may be effective in generating short-term engagement, they risk becoming repetitive or losing their appeal over time. More critically, they may fail to foster sustained engagement or promote intrinsic motivation, which are essential for long-term behavioral change and integration into routine practice [11,12,28,76]. These extrinsic motivators are highly effective at attracting initial participation, but they might not support enduring involvement as well as intrinsic motivators. Therefore, in the design of GDTs, striking a balance between extrinsic and intrinsic motivations is pivotal to maintaining engagement and fostering meaningful participation [28]. Regrettably, none of the studies analyzed in this review investigate the long-term impact of gamification or identify which specific gamification elements are most effective compared to others, limiting our ability to draw conclusions about the sustained benefits and potential limitations of GDTs.

Furthermore, although gamification is no longer a new concept in ICT, none of the studies reviewed addressed potential ethical issues. Although gamification is generally well received by HCWs, gamification may lead to unintended consequences, including addiction, cheating, or compromised data quality, especially when points, badges, or leaderboards overshadow the intended purpose [77,78]. For example, rewarding speed over accuracy may result in errors. Competition, one of the most common gamification elements adopted, fosters a spirit of healthy competition among HCWs [79]. However, it also carries the risk of demoralizing workers due to feelings of irrelevance and oppression by other users and of moving the focus excessively on measurable outcomes rather than quality [77]. To mitigate this, competition should promote teamwork through group-based formats rather than individual performance. Additionally, gamification raises ethical concerns related to user autonomy and consent [74].

Despite ethical considerations being increasingly recognized in ICTs [80], the ethical discourse within the gamification field remains underdeveloped [80-82]. Authors like Kim and Werbach [80] urge caution to avoid manipulative or harmful design practices.

### Strengths and Limitations

This study has 2 main strengths. First, by examining the current literature and available tools, we provided an initial overview of the potential effects of integrating gamified digital tools into the daily tasks of HCWs. Second, by conducting a scoping review to map the literature broadly, we have paved the way for future work to identify and select key outcomes for systematic reviews and meta-analyses in this domain more easily.

Our review also has certain limitations. First, it was challenging to identify studies that applied gamification to the daily tasks of HCWs, as many studies do not mention gamification explicitly. To locate potential studies, we thoroughly examined the methodologies and ICT interventions to pinpoint elements of gamification accurately. Second, most interventions researched and included in our review were pilots or case studies, characterized by small sample sizes and brief follow-ups. As a result, these studies have several limitations in evaluating long-term benefits that can affect their reliability and generalizability. Third, many studies do not rigorously describe or assess changes in motivation, behavior, or health outcomes. Fourth, given our goal to capture all relevant evidence and examples, we included a broad range of study designs, as is typical in a scoping review. Due to the rapid growth in digital health, prioritizing evidence based on quality criteria to capture scientifically based findings is challenging. Therefore, we applied the criteria developed by Sardi et al [11] to assess publications focusing on GDTs, ensuring the inclusion of evidence obtained through the most scientifically sound methodologies. Fifth, although our search was not limited to English, we were unable to find evidence in Spanish, French, or Italian. Lastly, only 1 author performed the data extraction, which may have introduced errors or biases. However, to ensure the accuracy of data extraction, all authors discussed which information should be extracted before the extraction process began. Subsequently, 2 authors carefully reviewed the extracted data to verify their accuracy.

### Future Directions

Our study highlighted that no study to date has directly compared gamified digital tools with their nongamified counterparts to assess their impact on HCWs’ daily tasks. Future research would benefit from adopting a 3-arm study design to better isolate the effects of gamification: (1) a control group without any digital tool, (2) a group using a nongamified digital tool, and (3) a group using a gamified digital tool. Furthermore, studies should incorporate longer follow-up periods and larger sample sizes to overcome the evident limitations identified in this scoping review, namely small sample sizes, lack of comparative groups, and short study durations. Extended follow-ups and broader participant inclusion would not only strengthen the robustness of findings but also allow the detection of potential tool misuse and ethical breaches that may emerge over time. Such an approach would enable a more comprehensive evaluation of whether gamification truly adds value beyond the use of digital tools alone and whether its effects are sustainable and ethically sound.

### Conclusion

The integration of GDTs into the daily workflows of HCWs holds significant potential to enhance engagement, motivation, and overall performance across a range of health-related tasks. However, this area remains largely underexplored, with limited empirical evidence to support its systematic implementation. This gap needs more robust comparative studies to provide precise insights into the benefits and limitations of GDTs.

Therefore, due to the limited evidence base, gamification remains less developed than other components of digital health programming, such as scalability, interoperability, or digital health landscape assessment, which are often supported by established frameworks and tools. Currently, there are no formal guidelines or standardized frameworks to assist developers and policymakers in effectively integrating GDTs into broader digital strategies.

Additionally, the lack of such guidance raises the risk of ethical misuse, such as manipulation, coercion, or unintended negative effects. Therefore, clear design principles and ethical safeguards are necessary to ensure that gamification boosts engagement without harming professional integrity or user autonomy.

## Supplementary material

10.2196/70480 Multimedia Appendix 1 PRISMA-ScR checklist.

10.2196/70480 Multimedia Appendix 2 Search strategy.

10.2196/70480 Multimedia Appendix 3 The quality assessment checklist and results.

## Abbreviations

AF: atrial fibrillation
CHW: community health care worker
CLABSI: Central Line-Associated Bloodstream Infections
EMR: electronic medical record
EPI: Expanded Programme on Immunization
GDT: gamified digital tools
HCW: health care worker
HIC: high-income country
ICT: information and communication technology
LMIC: low- and middle-income countries
PHC: primary health care
SAP: surgical antibiotic prophylaxis
