# Supplementary material for: Use of Gamified Digital Tools in Daily Tasks of Health Care Workers: Scoping Review
Source: JMIR Serious Games. 2025 Oct 20;13:e70480. doi: 10.2196/70480 (PMC12536924; doi:10.2196/70480)
Supplement: Multimedia Appendix 2 [file games-v13-e70480-s002.docx]

**Multimedia Appendix 2. Search strategy.**

| PubMed | (("gamif*"[Title/Abstract] OR "game*"[Title/Abstract] OR "Gamification"[MeSH Terms] OR "video game*"[Title/Abstract] OR "videogame*"[Title/Abstract] OR "Social Media"[MeSH Terms] OR "video games"[MeSH Terms] OR "playful design"[Title/Abstract] OR "Motivational design"[Title/Abstract] OR "Edutainment"[Title/Abstract] OR "Mobile Applications"[MeSH Terms]) AND ("cell phone"[MeSH Terms] OR "cell phone*"[Title/Abstract] OR "cellphone*"[Title/Abstract] OR "Smartphone"[MeSH Terms] OR "smartphone*"[Title/Abstract] OR "telephone"[MeSH Terms] OR "laptop*"[Title/Abstract] OR "computer*"[Title/Abstract] OR "tablet*"[Title/Abstract] OR "text messaging"[MeSH Terms] OR "text messaging"[Title/Abstract] OR "sms"[Title/Abstract] OR "Reminder Systems"[MeSH Terms] OR "reminders*"[Title/Abstract] OR "telemedicine"[MeSH Terms] OR "telemedicine"[Title/Abstract] OR "Mobile Applications"[MeSH Terms] OR "software"[MeSH Terms] OR "software"[Title/Abstract] OR "decision support systems, clinical"[MeSH Terms] OR "clinical decision support system*"[Title/Abstract] OR "e-health"[Title/Abstract] OR "ehealth"[Title/Abstract] OR "m-health"[Title/Abstract] OR "mhealth"[Title/Abstract] OR "digital technology"[MeSH Terms] OR "digital*"[Title/Abstract] OR "digitizat*"[Title/Abstract] OR "digitize*"[Title/Abstract] OR "digitizing"[Title/Abstract] OR "Electronic Health Records"[MeSH Terms] OR "medical records systems, computerized"[MeSH Terms] OR "electronic health record*"[Title/Abstract] OR "Machine Learning"[MeSH Terms] OR "Machine Learning"[Title/Abstract] OR "artificial intelligence"[MeSH Terms] OR "artificial intelligence"[Title/Abstract] OR ("medical"[Title/Abstract] AND "health"[Title/Abstract] AND "record*"[Title/Abstract]) OR "DHIS"[Title/Abstract] OR "Registries"[MeSH Terms] OR "registr*"[Title/Abstract] OR "computers, handheld"[MeSH Terms] OR "Database Management Systems"[MeSH Terms] OR "app"[Title/Abstract] OR "apps"[Title/Abstract] OR "Internet"[MeSH Terms] OR "Medical Informatics Applications"[MeSH Terms] OR "therapy, computer assisted"[MeSH Terms]) AND ("health personnel"[MeSH Terms] OR "healthcare worker*"[All Fields] OR "health care professional*"[All Fields] OR "Health Manpower"[Title/Abstract] OR "health workforce"[MeSH Terms] OR "staff"[Title/Abstract] OR "community health workers"[MeSH Terms] OR "Physicians"[MeSH Terms] OR "Nurses"[MeSH Terms] OR "Pharmacists"[MeSH Terms] OR "clerk*"[All Fields])) AND (2010/1/1:2023/10/27[pdat])  Date of search: 2023/10/27 |
| --- | --- |
| Embase (Ovid) | 1. (gamification or game or videogame or video games or social media or playful design or motivational design or edutainment or mobile application or mobile app).mp. [mp=ti, ab, hw, tn, ot, dm, mf, dv, kf, fx, dq, cw, bt, nm, ox, px, rx, ui, sy, ux, mx] 2. (healthcare workers or health personnel or HCW or CHW or Community health workers or community health volunteers or health workforce or staff or physicians or nurses or pharmacists or clerk or doctors).mp. [mp=ti, ab, hw, tn, ot, dm, mf, dv, kf, fx, dq, cw, bt, nm, ox, px, rx, ui, sy, ux, mx] 3. 1 AND 2   Date of search: 2024/01/26 |
| Google scholar | allintitle: ("gamification" OR "game" OR "videogame" OR "video games" OR "social media" OR "playful design" OR "Motivational design" OR "edutainment" OR "mobile application" OR "mobile app") AND (“healthcare workers” OR “health personnel” OR “HCW” OR “CHW” OR “Community health workers” OR “community health volunteers” OR “health workforce” OR “staff” OR “physicians” OR “nurses” OR “pharmacists” OR “Clerk” OR “doctors”)  Date of search: 2024/01/24 |
