# Supplementary material for: Use of Gamified Digital Tools in Daily Tasks of Health Care Workers: Scoping Review
Source: JMIR Serious Games. 2025 Oct 20;13:e70480. doi: 10.2196/70480 (PMC12536924; doi:10.2196/70480)
Supplement: Multimedia Appendix 3 [file games-v13-e70480-s003.docx]

**Multimedia Appendix 3. The quality assessment checklist and results.**

According to Sardi et al. [11]

| Quality assessment questions | Answer |
| --- | --- |
| Q1. Does the paper present a detailed description  of the game elements employed? * | “Yes (+1)”, No “(+0)” and “Partially (+0.5)”. |
| Q2. Does the study present empirical results? | “Yes (+1)” and No “(+0)”. |
| Q3. Are the limitations of gamification addressed explicitly? | “Yes (+1)” and No “(+0)”. |
| Q4. Does the paper discuss the benefits of gamification? | “Yes (+1)” and No “(+0)”. |
| Q5. Has the study been published in a relevant journal or conference proceedings? ** | for conferences, workshops, and symposia  (https://portal.core.edu.au/conf-ranks/):   - (+1.5) if it is ranked CORE A - (+1) If it is ranked CORE B - (+0.5) If it is ranked CORE C - (+0) If it is not in the CORE ranking   For journals (<https://www.scimagojr.com>):  o (+2) If it is ranked Q1  o (+1.5) If it is ranked Q2  o (+1) If it is ranked Q3 or Q4  o (+0) if it has no JCR ranking  for others: (+0). |

*Question Q1 scores partially when the paper does not provide details about the game elements employed in the application.

**The differentiation made as regards the score for question Q5 is owing to the fact that it is usually more complicated to publish in ranked journals than in conferences or symposia.

**Quality Assessment Results**

| Study, Year | Quality assessment | | | | | | |
| --- | --- | --- | --- | --- | --- | --- | --- |
|  | **Q1** | **Q2** | **Q3** | **Q4** | **Q5** | **Total** | **Rank** |
| Luedtke et al. [57], 2023 | 1 | 1 | 0 | 1 | 2 | 5 | High |
| Finette et al. [62], 2019 | 0.5 | 1 | 0 | 0 | 2 | 3.5 | Medium |
| Shah et al. [58], 2019 | 0 | 1 | 0 | 0 | 0 | 1 | Poor |
| Zaidi et al. [59], 2020 | 0.5 | 1 | 0 | 1 | 2 | 4.5 | High |
| Mckeown et al. [51], 2016 | 1 | 1 | 0 | 0 | 1 | 3 | Medium |
| Orchard et al. [52], 2019 | 0 | 1 | 0 | 1 | 2 | 4 | High |
| Orwoll et al. [53], 2018 | 1 | 1 | 0 | 1 | 1.5 | 4.5 | High |
| Wenner et al. [60], 2014 | 1 | 0 | 1 | 0 | 0 | 2 | Poor |
| Little et al. [61], 2013 | 0.5 | 1 | 0 | 0 | 2 | 3.5 | Medium |
| Owens et al. [55], 2018 | 0 | 1 | 0 | 1 | 0 | 2 | Poor |
| Collins et al. [54], 2013 | 1 | 1 | 0 | 1 | 0 | 3 | Medium |
| Marques et al. [56], 2017 | 1 | 0 | 1 | 1 | 2 | 5 | High |
| Average = 3.416 |  |  |  |  |  |  |  |
